# Supplementary material for: Comparative Genomic Analysis and Metabolic Potential Profiling of a Novel Culinary-Medicinal Mushroom, Hericium rajendrae (Basidiomycota)
Source: J Fungi (Basel). 2023 Oct 15;9(10):1018. doi: 10.3390/jof9101018 (PMC10608310; doi:10.3390/jof9101018)
Supplement: Supplementary file 1 [file jof-09-01018-s001.zip › Supplemental Material.pdf]

Comparative genomic analysis and metabolic potential profiling of culinary-medicinal mushroom *Hericium rajendrae* (Basidiomycota)

Jing Wei[123], Min Cheng[1], Jianfang Zhu[2], Yilin Zhang[1], Kun Cui[2], Xuejun Wang[1] *, Jianzhao Qi[12] *

1 Shangluo Key Research Laboratory of Standardized Planting & Quality Improvement of Bulk Chinese Medicinal Materials, College of Biology Pharmacy & Food Engineering, Shangluo University, Shangluo 726000, China;

2 Shaanxi Key Laboratory of Natural Products & Chemical Biology, College of Chemistry & Pharmacy, North-west A&F University, 3 Taicheng Road, Xianyang 712100, China;

3 Qinba Mountains of Bio-Resource Collaborative Innovation Center of Southern Shaanxi Province, Hanzhong 7230010, China.

* Correspondence: xuejunwangd@163.com (X.W.); qjz@nwafu.edu.cn (J.Q.)

General isolation process 1
Table S1. Estimation of genome size of *H. rajendrae* NPCB-A08. 2
Table S2. Statistics of Illumina NovaSeq sequencing data mapping of *H. rajendrae* NPCB-A08 genome. 3
Table S3. Statistics of Oxford Nanopore PromethION sequencing data volume of *H. rajendrae* NPCB-A08 genome. 4
Table S4. Statistics on assembly results. 5
Table S5. Statistics of Illumina NovaSeq sequencing data mapping and BUSCO evaluation of *H. rajendrae* NPCB-A08 genome. 6
Table S6. Statistics of *H. rajendrae* NPCB-A08 protein-coding gene annotation. 7
Table S7. Statistics of non-coding RNA annotation results in *H. rajendrae* NPCB-A08 genome. 8
Table S8. Annotation Statistics of coding gene from the *H. rajendrae* NPCB-A08 genome. 9
Table S9. Species genomes for evolutionary inference and gene family variation. 10
Table S10. The composition of CAZymes of edible mushrooms. 11
Table S11. Number and relative abundance of SSRs identified in the genus *Hericium*. 12
Table S12. Terpenoid biosynthesis related enzymes in *H. rajendrae* NPCB-A08 genome. 13
Table S13. ^{1}H and ^{13}C NMR Spectroscopic data of compounds 1, 4, 5, and 7. 14
Figure S1. ITS alignment of the strain NPCB-A08. 15
Figure S2. Species distribution of the top 10 homologous sequences using the annotation results from the Nr database. 16
Figure S3. Statistical map of functional annotation classification based on GO database 17
Figure S4. Statistical Chart of COG Functional Annotated Classification. 18
Figure S5. KEGG Pathway Functional Classification Chart. 19
Figure S6. Domain annotation based on the Pfam database. 20
Figure S7. Comparative genome and genomic collinearity analysis among the genus *Hericium*. 21
Figure S8. P450s Cluster analysis of the strain NPCB-A08 and other Basidiomycetes. 22
Figure S9. FPPs Cluster analysis of the strain NPCB-A08 and other Basidiomycetes. 23
Figure S10. Molecular network analysis of metabolites from the mycelium and fruiting bodies of the strain NPCB-A08. 24
Figure S11. The LC-ESI-HRMS and LC-ESI-HRMS/MS spectrums of isolates from the strain NPCB A08. 25
Figure S12. The NMR spectrums of compound 1 (MeOD) from the strain NPCB A08 28
Figure S13. The NMR spectrums of compound 4 (MeOD) from the strain NPCB A08 29
Figure S14. The NMR spectrums of compound 5 (MeOD) from the strain NPCB A08 30
Figure S15. The NMR spectrums of compound 7 (MeOD) from the strain NPCB A08 31
Reference 32

General isolation process

The resulting extracts were then separated and purified with different types of chromatography, including silica gel, reversed-phase C18 column, and Sephadex LH-20. A detailed separation process is as follows: Totals of 12 compounds were separated on a semipreparative HPLC equipped with a Hypersil BDS C18 column (4.6 mm × 250 mm; 10.0 mm × 250 mm). Column chromatography (CC) was carried out using silica gel (100-200 mesh and 300-400 mesh, Qingdao Marine Chemical Ltd., People's Republic of China), Sephadex LH-20 (GE Healthcare), and reversed-phase C18 silica gel (RP-18, GE Healthcare). Thin layer chromatography used precoated plates of silica gel 60 F_{254}, and spots were visualized under UV light (254 and 365 nm) or by spraying with vanilin-H_2SO_4 10% solution and heating for two minutes. Concentration was performed with a Büchi Rotavapor R-101.

The crude extract was applied to a silica gel column eluted with a gradient of $CHCl_3$–MeOH (100:1 200 mL, 50:1 200 mL, 25:1, 200 mL, 10:1 200 mL, 5:1 200 mL, 2:1 200 mL and MeOH 100 mL) to give seven fractions (Fr. F1–F7). F-2 was separated by RP-18 (MeOH–H_2O, 10–100%) to give five fractions (F-2-1–F-2-5). Fraction F-2-3 was subjected to Sephadex LH-20 (MeOH) and further purified by semipreparative HPLC (70%, MeOH–H_2O, 2 mL/min) to yield compound **4** (t_R = 14 min, 12.0 mg). F-2-4 was purified by semipreparative HPLC (50%, MeOH–H_2O, 2 mL/min) to yield compound **11** (t_R = 15 min, 2.0 mg). F-2-5 was purified by semipreparative HPLC (53%, MeOH–H_2O, 2 mL/min) to yield compound 8 (t_R = 21 min, 0.8 mg).

F-3 was separated by RP-18 (10% –100%, MeOH–H_2O) to obtain five subfractions (F-3-1–F-3-5). F-3-3 was purified by silica gel CC ($CHCl_3$–MeOH, 10:1) followed by Sephadex LH-20 (MeOH) to obtain three Fractions (F-3-3-1—F-3-3-3). Fraction F-3-3-1 was applied to silica gel CC (petroleum ether–acetone, 6:1) to afford compound **9** (1.5 mg). F-3-3-2 was purified by semipreparative HPLC (60%, MeOH–H_2O, 2 mL/min) to yield compound **10** (t_R = 16 min, 4.0 mg). F-3-3-3 was purified by semipreparative HPLC (70%, MeOH–H_2O, 2 mL/min) to yield compound **12** (t_R = 15 min, 1.0 mg).

F-4 was separated by RP-18 (10% –100%, MeOH–H_2O) to obtain five subfractions (F-4-1–F-4-5). F-4-2 was purified by silica gel CC ($CHCl_3$–MeOH, 15:1) followed by Sephadex LH-20 (MeOH) to obtain three Fractions (F-4-2-1—F-4-2-4). Fraction F-4-2-1 was purified by semipreparative HPLC (60%, MeOH–H2O, 2 mL/min) to yield compound **5** (t_R = 18 min, 12.0 mg). F-4-2-2 was purified by semipreparative HPLC (65%, MeOH–H_2O, 2 mL/min) to yield compound **3** (t_R = 20 min, 1.9 mg).

F-5 was separated by RP-18 (10% –100%, MeOH–H_2O) to obtain five subfractions (F-5-1–F-5-5). F-5-1 was purified by silica gel CC ($CHCl_3$–MeOH, 20:1) followed by Sephadex LH-20 (MeOH) to obtain three Fractions (F-5-1-1—F-5-1-4). Fraction F-5-1-1 was purified by semipreparative HPLC (60%, MeOH–H_2O, 2 mL/min) to yield compound **1** (t_R = 22 min, 15.0 mg). F-5-1-2 was purified by semipreparative HPLC (55%, MeOH–H_2O, 2 mL/min) to yield compound 2 (t_R = 20 min, 2.5 mg). F-5-2 was purified by silica gel CC ($CHCl_3$–MeOH, 25:1) followed by Sephadex LH-20 (MeOH) to obtain three Fractions (F-5-2-1—F-5-2-3). Fraction F-5-2-1 was purified by semipreparative HPLC (65%, MeOH–H_2O, 2 mL/min) to yield compound **6** (t_R = 18 min, 5.0 mg). F-5-2-2 was purified by semipreparative HPLC (54%, MeOH–H_2O, 2 mL/min) to yield compound **7** (t_R = 20 min, 10.0 mg).

Table S1. Estimation of genome size of *H. rajendrae* NPCB-A08.

Item	Number
Heterozygosity	3.612%
Genome Haploid Length	46,016,108 bp
Genome Repeat Length	18,531,666 bp
Genome Unique Length	27,484,441 bp
Model Fit	94.091%
Read Error Rate	0.059%

Genome size was estimated using genomescope (version 1.0.0) software.

Table S2. Statistics of Illumina NovaSeq sequencing data mapping of *H. rajendrae* NPCB-A08 genome.

Sample_name	Total_reads	Total_bases	GC_content	Q20	Q30
rawdata	41,771,188	6,265,678,200	52.54%	97.55%	93.26%
cleandata	41,771,008	6,265,651,200	52.54%	97.55%	93.26%

Sample name is data type; **Total_reads** is sequencing reads number; **Total_bases** is total sequencing base number; **GC_Content** is G/C base number as a percentage of total base number; **Q20**、**Q30** respectively are Phred value greater than 20、30 base as a percentage of total base.

Table S3. Statistics of Oxford Nanopore PromethION sequencing data volume of *H. rajendrae* NPCB-A08 genome.

Rank	Flag	TotalBase	TotalReads	MaxLen	AvgLen	N50	L50	N90	L90	meanQ
>0	all	12,185,359,000	2,522,073	142,428	4,831.48	9,230	302,680	2,234	1,452,695	12.13
>0	pass	11,887,921,275	2,455,505	142,428	4,841.33	9,236	295,271	2,238	1,416,066	12.29
>0	fail	297,437,725	66,568	111,084	4,468.17	9,004	7,412	2,100	36,680	6.17
>5000	all	8,198,403,457	619,884	142,428	13,225.7	17,176	142,086	6,281	473,145	12.11
>5000	pass	8,001,403,187	605,123	142,428	13,222.77	17,171	138,740	6,280	461,905	12.26
>5000	fail	197,000,270	14,761	111,084	13,345.99	17,429	3,348	6,326	11,241	6.18
>10000	all	5,835,706,124	275,920	142,428	21,149.99	23,841	83,665	12,019	222,603	12.04
>10000	pass	5,694,739,811	269,326	142,428	21,144.41	23,835	81,688	12,017	217,296	12.18
>10000	fail	140,966,313	6,594	111,084	21,377.96	24,085	1,978	12,098	5,309	6.19
>50000	all	411,941,772	7,068	142,428	58,282.65	56,548	3,173	51,026	6,252	11.86
>50000	pass	400,287,964	6,876	142,428	58,215.23	56,458	3,089	51,016	6,083	12.01
>50000	fail	11,653,808	192	111,084	60,696.91	59,118	85	51,222	170	6.28
>100000	all	4,309,814	39	142,428	110,508.05	108,906	19	101,908	35	8.98
>100000	pass	3,987,855	36	142,428	110,773.75	109,240	17	101,866	33	9.20
>100000	fail	321,959	3	111,084	107,319.66	108,906	2	101,969	3	6.33

Rank is the gradient of data length, >0 is that all data; Flag is data type, all is all sequencing data, pass is effective sequencing data, fail is filtration data; Total Base is the number of base; Total Reads is the number of reads; MaxLen is maximum length of data; AvgLen is average length of data; N50 is N50 of data, all reads are summed in order from long to short, and when additive length up to half of all reads total length, the last read length added is N50; L50 is L50 of data, all reads are accumulated in turn ranking in order from long to short, when additive length up to half of all reads total length, the number of sequences is L50; N90 is N90 of data, algorithm the same as N50; L90 is L90 of data, algorithm the same as L50; meanQ is mean quality value.

Table S4. Statistics on assembly results.

Item	Value
Total_length(bp)	46,767,965
Total_length_withoutN (bp)	46,767,965
Total_number	19
GC_content (%)	52.57
N50(bp)	3,238,877
N90(bp)	2,017,740
Average(bp)	2,461,471.84
Median(bp)	2,306,246.00
Min(bp)	191,581
Max(bp)	5,307,752

Table S5. Statistics of Illumina NovaSeq sequencing data mapping and BUSCO evaluation of *H. rajendrae* NPCB-A08 genome.

Type	Namber
map_rate	92.61%
Average_depth	118.67
Coverage	99.95%

map_rate is the second-generation data comparison rate, and Average_depth is the average coverage depth.

Item	Number	Percent (%)
Complete BUSCOs (C)	694	91.6
Complete and single-copy BUSCOs (S)	675	89.1
Complete and duplicated BUSCOs (D)	19	2.5
Fragmented BUSCOs (F)	26	3.4
Missing BUSCOs (M)	38	5.0
Total BUSCO groups searched	758	100.0

Single-copy for single-copy BUSCOs; duplicated for multicopy BUSCOs; Fragmented for fragmented BUSCOs; Missing for missing BUSCOs. The predicted genes were assessed for completeness using the BUSCO software (version: 4.1.4) based on the fungi database (fungi_odb10).

Table S6. Statistics of *H. rajendrae* NPCB-A08 protein-coding gene annotation.

Item	Count	Percentage
All	13,418	100%
Annotation	11,716	87.31%
Uniprot	5,555	41.40%
Pfam	8,251	61.49%
Refseq	3,969	29.58%
Nr	11,628	86.66%
Interproscan	8,246	61.45%
GO	5,512	41.08%
KEGG	4,032	30.05%
Pathway	2,502	18.65%
COG	1,049	7.82%

Annotation is the gene with at least one annotation; Uniprot is the gene annotated to the Uniprot database; Pfam is the gene that is annotated to the Pfam database; Refseq is the gene that is annotated to the Refseq database; Nr is the gene that is annotated to the Nr database; Interproscan is the gene that is annotated to the Interproscan GO is the gene annotated to the GO database; KEGG is the gene that is annotated to the KEGG database; Pathway is the gene that is annotated to the KEGG Pathway database; COG is the gene that is annotated to the COG database.

Table S7. Statistics of non-coding RNA annotation results in *H. rajendrae* NPCB-A08 genome.

Class	number	totalLen(bp)	meanLen(bp)
rRNA	38	53,309	1,402
sRNA	1	340	340
snRNA	29	3,771	130
tRNA	136	12,281	90

rRNA is ribosomal RNA; tRNA is transport RNA; sRNA is small regulatory RNA; snRNA is nucleolar small RNA. **totalLen** and **meanLen** are the total length and mean length.

Table S8. Annotation Statistics of coding gene from the *H. rajendrae* NPCB-A08 genome.

Type	Number
Total number of gene	13,418
Average of mRNA length	1,715.42
Average of cds length	1,273.36
Average of exon number	6.97
Average of exon length	182.74
Average of intron length	74.07
Total number of exon	93,499
Total number of intron	80,081
Total intron length	5,931,281

the total number of gene is the total number of genes; the average of mRNA_length is the average length of mRNA; the average of cds_length is the average length of CDS; the average of exon_number is the average number of exons per gene. the average of exon_length is the average exon length; the average of intron_length is the average intron length; the total number of exon is the total number of exons; the total number of intron is the total number of introns; the total intron length is the total intron length.

Table S9. Species genomes for evolutionary inference and gene family variation.

Entry	URL
Ustilago maydis	https://www.ncbi.nlm.nih.gov/datasets/genome/GCF_000328475.2/
Tremella mesenterica	https://www.ncbi.nlm.nih.gov/datasets/genome/GCF_000271645.1/
Rickenella mellea	https://www.ncbi.nlm.nih.gov/datasets/genome/GCA_004355085.1/
Phellinidium pouzarii	https://www.ncbi.nlm.nih.gov/datasets/genome/GCA_004802695.1/
Phellinus noxius	https://genome.jgi.doe.gov/portal/Pheno1/Pheno1.download.html
Porodaedalea chrysoloma	https://genome.jgi.doe.gov/portal/Porchr1/Porchr1.download.html
Sanghuangporus baumii	https://www.ncbi.nlm.nih.gov/datasets/genome/GCA_001481415.2/
Inonotus hispidus	https://www.ncbi.nlm.nih.gov/datasets/genome/GCA_024712875.1/
Suillus brevipes	https://www.ncbi.nlm.nih.gov/datasets/genome/GCA_011800875.2/
Boletus edulis	https://www.ncbi.nlm.nih.gov/datasets/genome/GCA_015179015.1/
Paxillus involutus	https://www.ncbi.nlm.nih.gov/datasets/genome/GCA_000827475.1/
Pteruala gracilis	https://www.ncbi.nlm.nih.gov/datasets/genome/GCA_004369125.1/
Pleurotus ostreatus	https://www.ncbi.nlm.nih.gov/datasets/genome/GCF_014466165.1/
Schizophyllum commune	https://www.ncbi.nlm.nih.gov/datasets/genome/GCF_000143185.2/
Lentinula edodes	https://www.ncbi.nlm.nih.gov/datasets/genome/GCF_021015755.1/
Oudemansiella raphanipes	ftp://www.mushroomlab.cn
Armillaria mellea	https://www.ncbi.nlm.nih.gov/datasets/genome/GCA_030407055.1/
Pluteus cervinus	https://www.ncbi.nlm.nih.gov/datasets/genome/GCA_004369065.1/
Amanita muscaria Koide	https://www.ncbi.nlm.nih.gov/datasets/genome/GCA_000827485.1/
Tricholoma matsutake	https://www.ncbi.nlm.nih.gov/datasets/genome/GCA_026075535.2/
Lyophyllum atratum	https://www.ncbi.nlm.nih.gov/datasets/genome/GCA_014905825.1/
Lyophyllum decastes	https://www.ncbi.nlm.nih.gov/datasets/genome/GCA_025854295.1/
Agaricus bitorquis	https://www.ncbi.nlm.nih.gov/datasets/genome/GCA_030246685.1/
Cortinarius glaucopus	https://www.ncbi.nlm.nih.gov/datasets/genome/GCA_015039465.1/
Cyclocybe aegerita	https://www.ncbi.nlm.nih.gov/datasets/genome/GCA_902728275.1/
Psilocybe cubensis	https://www.ncbi.nlm.nih.gov/datasets/genome/GCF_017499595.1/
Stropharia rugosoannulata	https://www.ncbi.nlm.nih.gov/datasets/genome/GCA_028532985.1/
Gloeophyllum trabeum	https://www.ncbi.nlm.nih.gov/datasets/genome/GCF_000344685.1/
Cristinia sonorae	https://genome.jgi.doe.gov/portal/Crison1/Crison1.download.html
Ganoderma sinense	https://www.ncbi.nlm.nih.gov/datasets/genome/GCA_002760635.1/
Grifola frondosa	https://www.ncbi.nlm.nih.gov/datasets/genome/GCA_001683735.1/
Gelatoporia subvermispora	https://www.ncbi.nlm.nih.gov/datasets/genome/GCA_000320605.2/
Sparassis crispa	https://www.ncbi.nlm.nih.gov/datasets/genome/GCF_003851025.1/
Wolfiporia cocos	https://genome.jgi.doe.gov/portal/Wolco1/Wolco1.download.html
Laetiporus sulphureus	https://www.ncbi.nlm.nih.gov/datasets/genome/GCF_001632365.1/
Stereum hirsutum	https://www.ncbi.nlm.nih.gov/datasets/genome/GCF_000264905.1/
Lactarius deliciosus	https://www.ncbi.nlm.nih.gov/datasets/genome/GCA_021525775.1/
Hericium coralloides FP-101451	https://genome.jgi.doe.gov/portal/Hercor1/Hercor1.download.html
Hericium erinaceus CS-4	https://www.ncbi.nlm.nih.gov/datasets/genome/GCA_006506795.2/

Table S10. The composition of CAZymes of edible mushrooms.

Species	AA	CBM	CE	GH	GT	PL
Agaricus bitorquis BH01	162	14	35	186	60	12
Agaricus bisporus var bisporus	95	13	34	177	55	13
Armillaria mellea ELDO17	149	32	46	275	79	29
Boletus edulis BED1	54	10	17	149	79	4
Cordyceps militaris CM01	58	8	13	159	87	4
Cyclocybe aegerita AAE3	112	20	32	214	65	18
Grifola frondosa 9006-11	86	3	20	171	62	11
Hericium rajendrae NPCB A08	87	7	23	189	65	9
Hericium coralloides FP-101451	87	6	27	175	63	10
Hericium erinaceus CS 4	83	7	26	162	60	8
*Hypsizygus marmoreus*51987-8	123	18	23	217	73	22
Lactarius deliciosus EDB83	94	7	15	125	83	8
Lentinula edodes Le (Bin) 0899 s	92	16	33	247	72	13
Lyophyllum decastes LRG-d1-5	124	27	37	236	71	26
Morchella conica CCBAS932	76	8	27	176	66	22
Morchella importuna M04M26	74	6	26	173	65	23
Morchella sextelata SCLS	79	8	29	177	71	23
Morchella snyderi CBS 144464	72	8	27	172	68	20
Oudemansiella raphanipes CGG-A	171	19	43	326	85	31
Paxillus ammoniavirescens Pou09	54	4	13	120	59	8
Paxillus involutus ATCC 200175	61	7	15	167	74	10
Pleurotus djamor MPG-05	250	95	47	387	88	47
Pleurotus eryngii ATCC 90797	117	21	28	229	69	35
Pleurotus ostreatus PC9	141	40	36	232	65	29
Sparassis crispa SCP 1.1	33	3	12	128	59	6
Stropharia rugosoannulata A15	148	15	39	213	70	18
Tremella mesenterica ATCC 28783	15	3	12	74	66	5
Tremella mesenterica Fries	16	4	13	72	67	5
Tricholoma matsutake 945	67	7	15	138	71	9
Tuber melanosporum Mel28	38	3	12	83	59	3
Ustilago maydis 521	30	0	17	105	65	2
Volvariella volvacea WC 439	136	33	39	270	68	30
Auricularia subglabra TFB-10046 SS5	143	23	74	364	68	31

Table S11. Number and relative abundance of SSRs identified in the genus *Hericium*.

	Motif	No.	Percentage (%)	Length overall(bp)	Average Length(bp)	No. motifs type	Longest pattern
***H. erinaceus* CS_4**	Monomer	272	19.43%	4216	15.5	4	$(G)_{39}$
	Dimer	230	16.43%	2972	12.9	12	$(TA)_{14}$
	Trimer	695	49.64%	12438	17.9	57	$(TCA)_{39}$
	Tetramer	76	5.43%	1772	23.3	35	$(GCTG)_{13}$
	Pentamer	67	4.79%	2565	38.2	34	$(GTTGG)_{30}$
	Hexamer	60	4.29%	2352	39.2	38	$(TAAAAT)_{11}$
	all SSRs	1400	100.00%	26315	18.8	180	$(GTTGG)_{30}$
	**Motif**	**No.**	**Percentage (%)**	**Length overall(bp)**	**Average Length(bp)**	**No. motifs type**	**Longest pattern**
***H. rajendrae* NPCB A08**	Monomer	257	17.75%	4275	16.6	4	$(T)_{55}$
	Dimer	346	23.90%	4504	13.1	12	$(AT)_{16}$
	Trimer	718	49.59%	12630	17.6	53	$(TAG)_{47}$
	Tetramer	73	5.04%	1680	23	37	$(GAAA)_{14}$
	Pentamer	26	1.80%	845	32.5	20	$(TGGGT)_{27}$
	Hexamer	28	1.93%	1254	44.7	25	$(TCATCC)_{39}$
	all SSRs	1448	100.00%	25188	17.3	151	$(TCATCC)_{39}$
	**Motif**	**No.**	**Percentage (%)**	**Length overall(bp)**	**Average Length(bp)**	**No. motifs type**	**Longest pattern**
***H. coralloides* FP-101451**	Monomer	103	10.89%	4926	47.8	4	$(A)_{1725}$
	Dimer	167	17.65%	2116	12.7	12	$(AG)_{11}$
	Trimer	591	62.47%	9891	16.7	48	$(TCC)_{13}$
	Tetramer	35	3.70%	756	21.6	22	$(GAAG)_{9}$
	Pentamer	25	2.64%	1115	44.6	19	$(GTTGG)_{27}$
	Hexamer	25	2.64%	1344	53.8	20	$(AAGGAG)_{42}$
	all SSRs	946	100.00%	20148	21.3	125	$(A)_{1725}$

Table S12. Terpenoid biosynthesis related enzymes in *H. rajendrae* NPCB-A08 genome.

Type	Entry	UniportKB database-based annotations	E-value	Indentify	Species	Accession Number
STS	g8004.t1	sesquiterpene synthase COP6	$2e^{-41}$	27.46%	*Coprinopsis cinerea*	A8NCK5.1
	g8005.t1	sesquiterpene synthase COP6	$2e^{-52}$	30.36%	*Coprinopsis cinerea*	A8NCK5.1
	g362 .t1	Sesquiterpene synthase 2	$2e^{-127}$	53.35%	*Postia placenta*	A0A348B780.1
	g468.t1	Sesquiterpene synthase 10	$2e^{-174}$	72.92%	*Postia placenta*	B8PD44.1
	g10218.t1	Sesquiterpene synthase 10	$3e^{-136}$	53.55%	*Postia placenta*	B8PD44.1
	g11367.t1	Sesquiterpene synthase Agr3	$2e^{-144}$	59.48%	*Cyclocybe aegerita*	A0A5Q0QU70.1
	g2467.t1	Sesquiterpene synthase 10	$2e^{-136}$	53.55%	*Postia placenta*	B8PD44.1
	g2512.t1	Sesquiterpene synthase 10	$1e^{-140}$	55.79%	*Postia placenta*	B8PD44.1
SQS	g11649.t1	squalene synthase	0	66.00%	*Ganoderma lucidum*	A0SJQ5.1
PSY	g12602.t1	NADH dehydrogenase (ubiquinone) complex I, assembly factor 6	$3e^{-71}$	41.16%	*Rattus norvegicus*	D3ZN43.1
LS	g12043.t1	Lanosterol synthase	0	67.32%	*Ganoderma lucidum*	D7NJ68.1

Table S13. ^{1}H and ^{13}C NMR Spectroscopic data of compounds 1, 4, 5, and 7.

	Erinacine A (1)[a]		Erinacine E (4)[a]		Erinacine F (5)[a]		Erinacine Z1 (7)[a]	
**No.**	δ_C	δ_H (m, *J* in Hz)	δ_C	δ_H (m, *J* in Hz)	δ_C	δ_H (m, *J* in Hz)	δ_C	δ_H (m, *J* in Hz)
**1**	39.6	1.65 m 1b<br>1.58 m 1a	40.1	1.68 m 1b<br>1.56 m 1a	39.2	1.64 m 2H	39.5	1.62 m 1b<br>1.54 m 1a
**2**	28.1	3.09 m 2b<br>2.48 m 2a	29.2	2.32 m 2H	29.0	2.30 m 2H	29.2	2.31 m 2b<br>2.29 m 2a
**3**	146.9		140.1		140.1		139.3	
**4**	143.7		138.7		139.3		138.8	
**5**	155.1		44.2	2.89 m	44.8	2.72 d (11.4)	41.5	1.98 d (11.4)
**6**	49.4		42.2		41.9		45.1	
**7**	29.6	2.55 m 7b<br>1.33 m 7a	28.5	1.76 m 7b<br>1.40 m 7a	28.4	1.83 m 7b<br>1.44 m 7a	31.1	1.79 m 7b<br>1.32 m 7a
**8**	37.6	1.63 m 8b<br>1.55 m 8a	38.1	1.56 m 2H	37.3	1.57 m 8b<br>1.54 m 8a	38.1	1.50 m 8b<br>1.43 m 8a
**9**	50.5		50.8		50.9		50.4	
**10**	121.3	5.80 d (8.0)	31.9	2.70 m 10b<br>2.60 m 10a	30.5	2.57 m 10b<br>2.44 m 10a	30.9	2.49 m 10b<br>1.83 m 10a
**11**	145.8	6.71 d (10.0)	124.0	5.63 m	121.8	5.52 m	74.0	4.56 m
**12**	140.5		142.6		140.2		140.5	
**13**	28.5	2.32 m 2H	43.9	3.11 m	53.2	3.18 d (12.7)	160.7	7.0 d (5.6)
**14**	85.5	3.54 d (6.4)	97.4	4.23 d (6.1)	91.6	4.28 d (10.7)	86.2	4.52 m
**15**	195.9	9.21 s	72.2	4.70 s	85.9	4.73 s	194.7	9.47 s
**16**	21.8	0.93 s 3H	17.3	0.96 s 3H	17.7	0.98 s 3H	16.5	1.02 s 3H
**17**	24.4	0.93 s 3H	25.0	1.09 s 3H	25.1	1.09 s 3H	24.9	0.93 s 3H
**18**	26.9	2.77 m	28.1	2.95 m	28.3	2.9 m	28.2	2.88 m
**19**	21.8	0.96 d (6.8) 3H	22.1	1.02 d (6.7) 3H	22.2	1.00 d (6.7) 3H	22.2	1.01 d (6.7) 3H
**20**	21.8	0.89 d (6.8) 3H	22.1	0.99 d (6.7) 3H	21.8	0.99 d (6.7) 3H	21.8	0.99 d (6.7) 3H
**1'**	107.1	4.19 d (7.3)	106.4	4.94 s	108.4	5.05 s	107.6	4.22 d (7.4)
**2'**	74.9	3.02 m	80.2		82.8		75.1	3.13 m
**3'**	77.8	3.17 m	75.8	3.90 s	71.0	3.77 m	77.9	3.26 m
**4'**	71.0	3.31 m	74.9		79.3		71.1	3.29 m
**5'**	66.5	3.67 dd (11.5, 5.1) 5'b; 3.00 m 5'a	66.5	3.92 m 5'b; 3.26 d (11.9) 5'a	64.3	3.69 m 2H	66.9	3.80 dd (11.4, 5.4)
**11-OMe**							56.8	3.23 s 3H
**References**	[1]		[2]		[2,3]		[4,5]	

a Measured in methanol-d4 at 125 MHz.

Sequences producing significant alignments Download Manage Columns Show 100

select all 100 sequences selected GenBank Graphics Distance tree of results

	Description	Max Score	Total Score	Query Cover	E value	Per. Ident	Accession
☑	Hericium rajendrae CAL 1717 ITS region; from TYPE material	1125	1125	96%	0.0	98.89%	NR_169943.1
☑	Hericium yumthangense BSHC KD-11-146 ITS region; from TYPE material	1044	1044	89%	0.0	98.97%	NR_155021.1
☑	Dentipellis tasmanica BJFC Dai 18767 ITS region; from TYPE material	593	593	76%	7e-170	88.51%	NR_168768.1
☑	Dentipellis coniferarum IFP 015823 ITS region; from TYPE material	508	508	76%	3e-144	85.52%	NR_132865.1
☑	Aleurodiscus thailandicus BJFC He4099 ITS region; from TYPE material	466	466	94%	2e-131	81.02%	NR_153577.1
☑	Heterobasidion araucariae CBS 743.94 ITS region; from TYPE material	436	436	94%	1e-122	80.03%	NR_138386.1
☑	Gloeocystidiellum bisporum CBS 961.96 ITS region; from TYPE material	433	433	81%	2e-121	82.10%	NR_160201.1
☑	Aleurodiscus tropicus BJFC 022332 ITS region; from TYPE material	433	433	73%	2e-121	83.57%	NR_158333.1
☑	Aleurodiscus bambusinus BJFC 023703 ITS region; from TYPE material	429	429	73%	2e-120	83.50%	NR_160568.1
☑	Amylostereum orientale BJFC He479 ITS region; from TYPE material	409	409	81%	3e-114	81.55%	NR_155020.1
☑	Neoaleurodiscus fujii TNM F22083 ITS region; from TYPE material	403	403	92%	1e-112	79.49%	NR_119723.1
☑	Veluticeps fasciculata BJFC Dai 15092 ITS region; from TYPE material	392	392	71%	3e-109	82.60%	NR_159011.1
☑	Aleurodiscus patagonicus MA Fungi 90714 ITS region; from TYPE material	390	390	63%	1e-108	84.07%	NR_164578.1
☑	Pseudowrightoporia hamata BJFC 2799 ITS region; from TYPE material	388	388	67%	4e-108	83.01%	NR_154619.1

Figure S1. ITS alignment of the strain NPCB-A08.

The ITS of strain NPCB-A08 was aligned to the nr database of NCBI (accessed on 10, September 18, 2020).

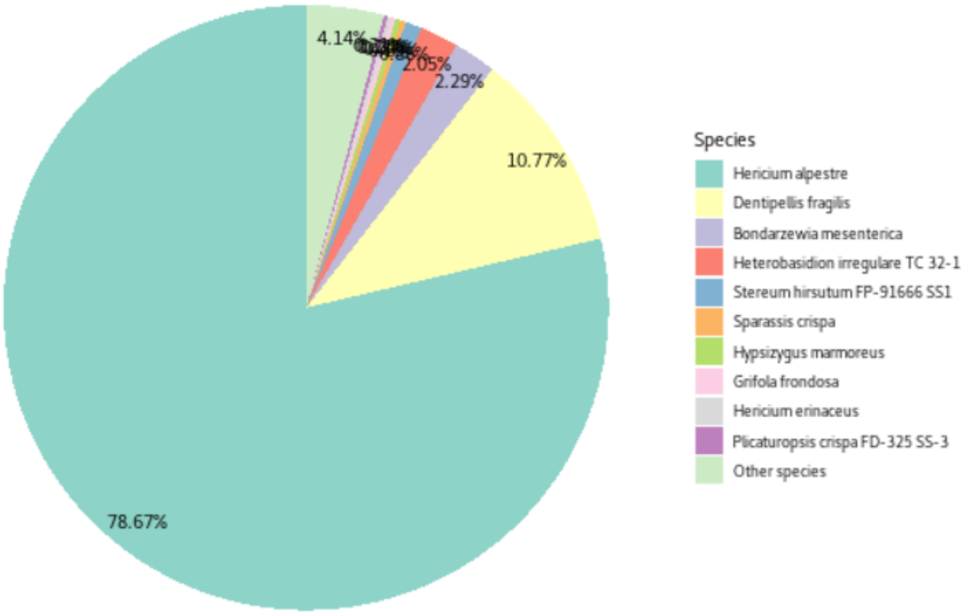

Figure S2. Species distribution of the top 10 homologous sequences using the annotation results from the Nr database.

According to the results of the Nr library match, the top 10 species were counted and the rest were classified as other species, and the distribution of these species was mapped according to their proportion.

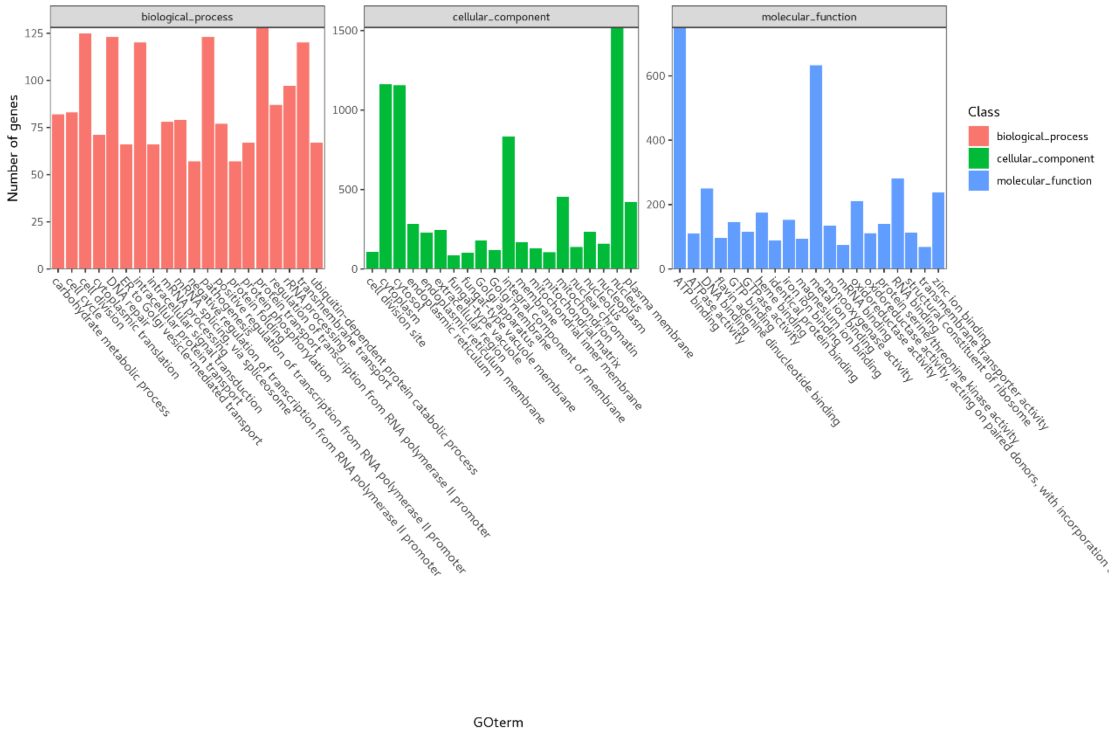

Figure S3. Statistical map of functional annotation classification based on GO database

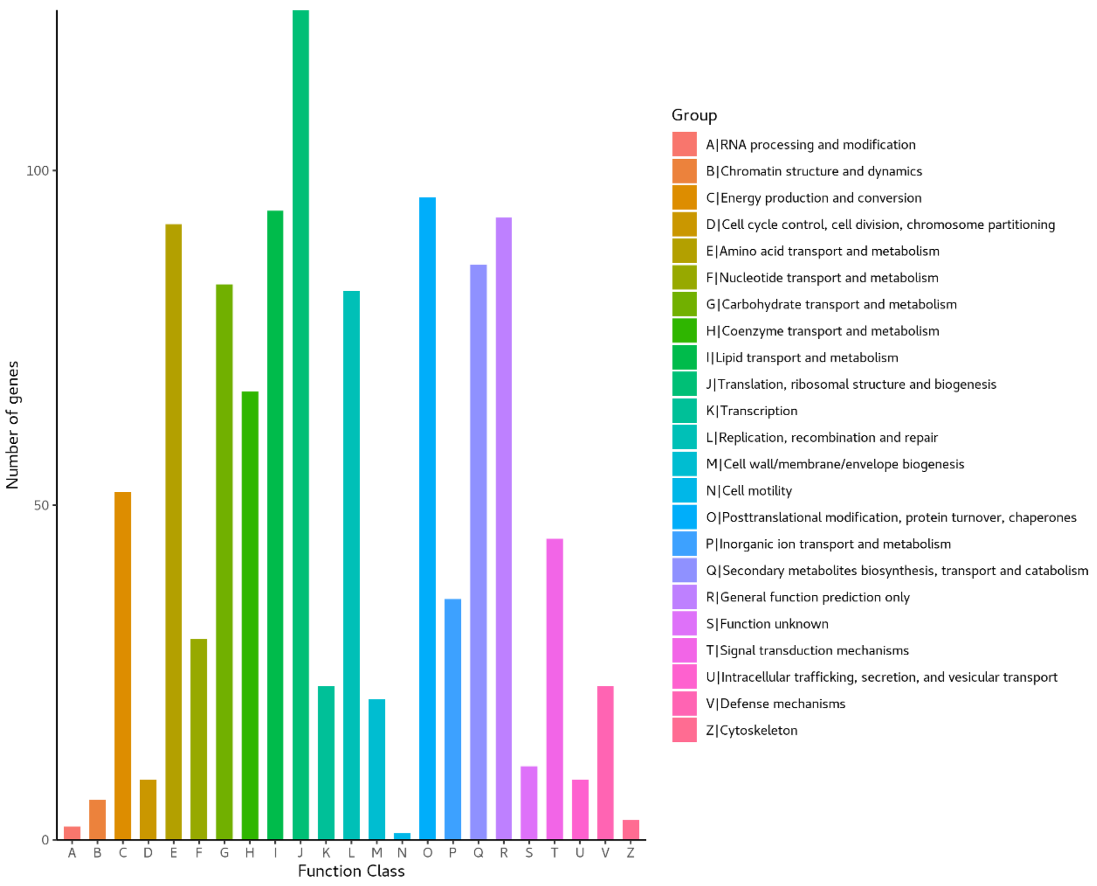

Figure S4. Statistical Chart of COG Functional Annotated Classification.

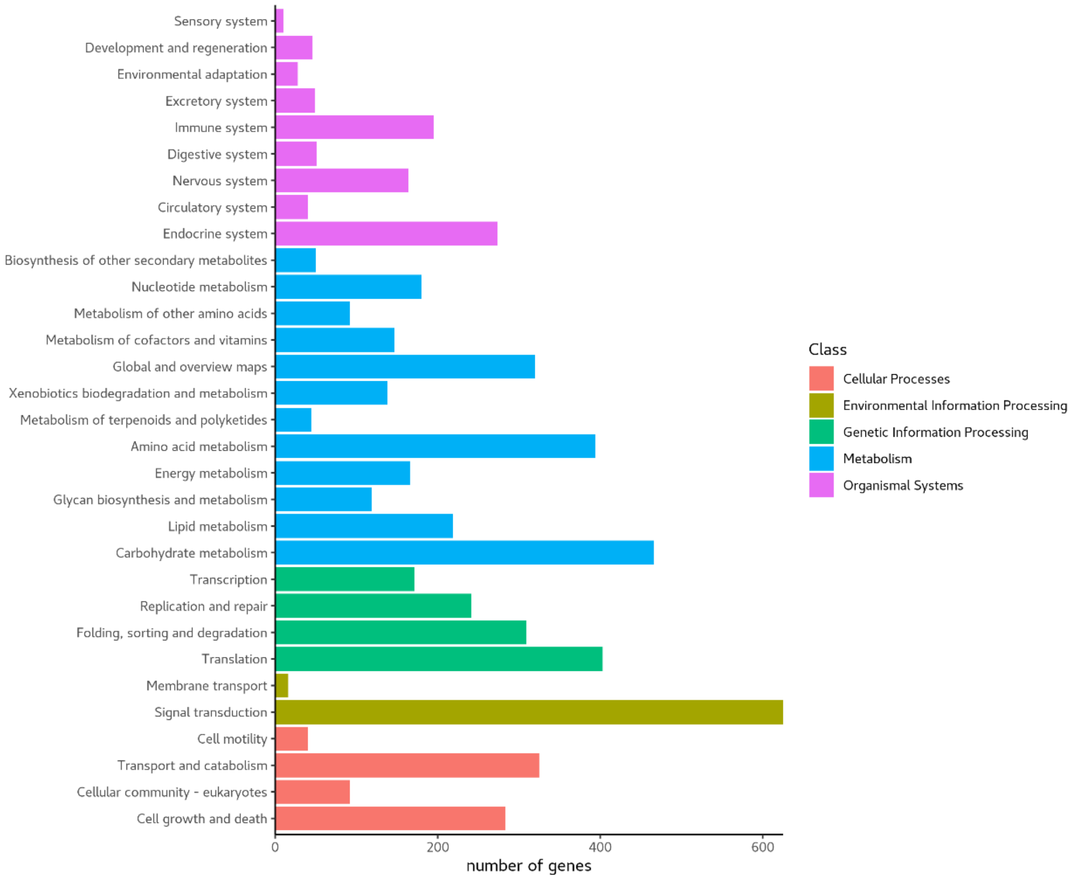

Figure S5. KEGG Pathway Functional Classification Chart.

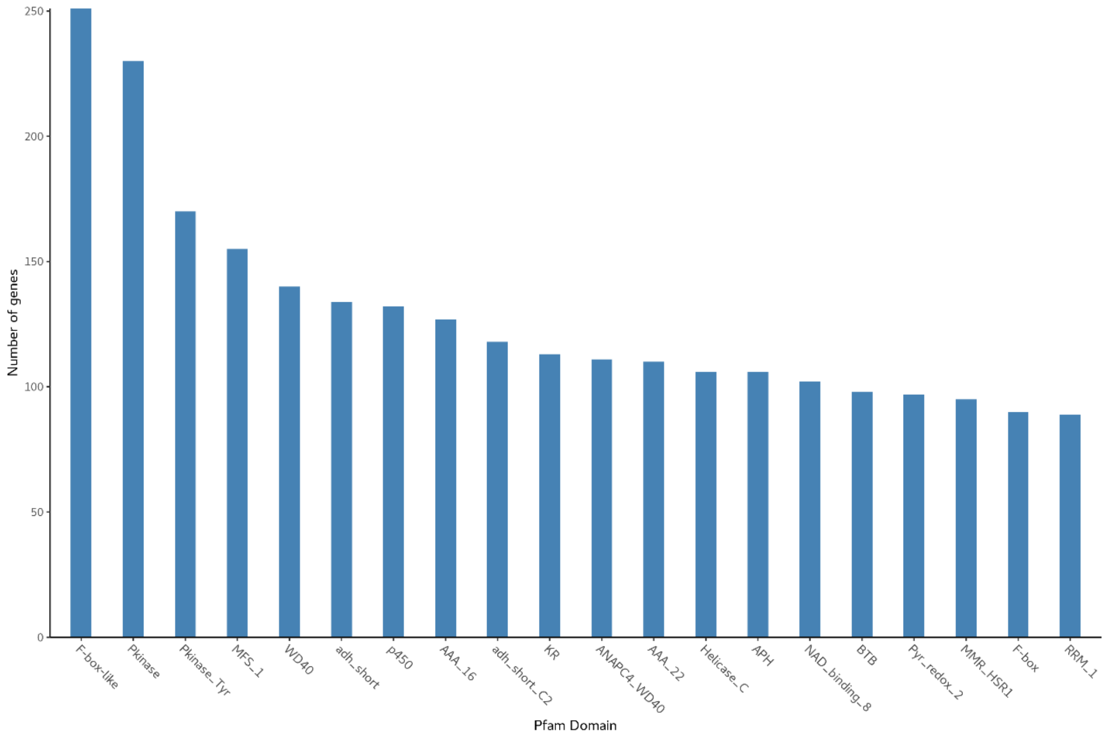

Figure S6. Domain annotation based on the Pfam database.

Statistical summaries of genes annotated for each domain are made and the top 20 most annotated domains are plotted.

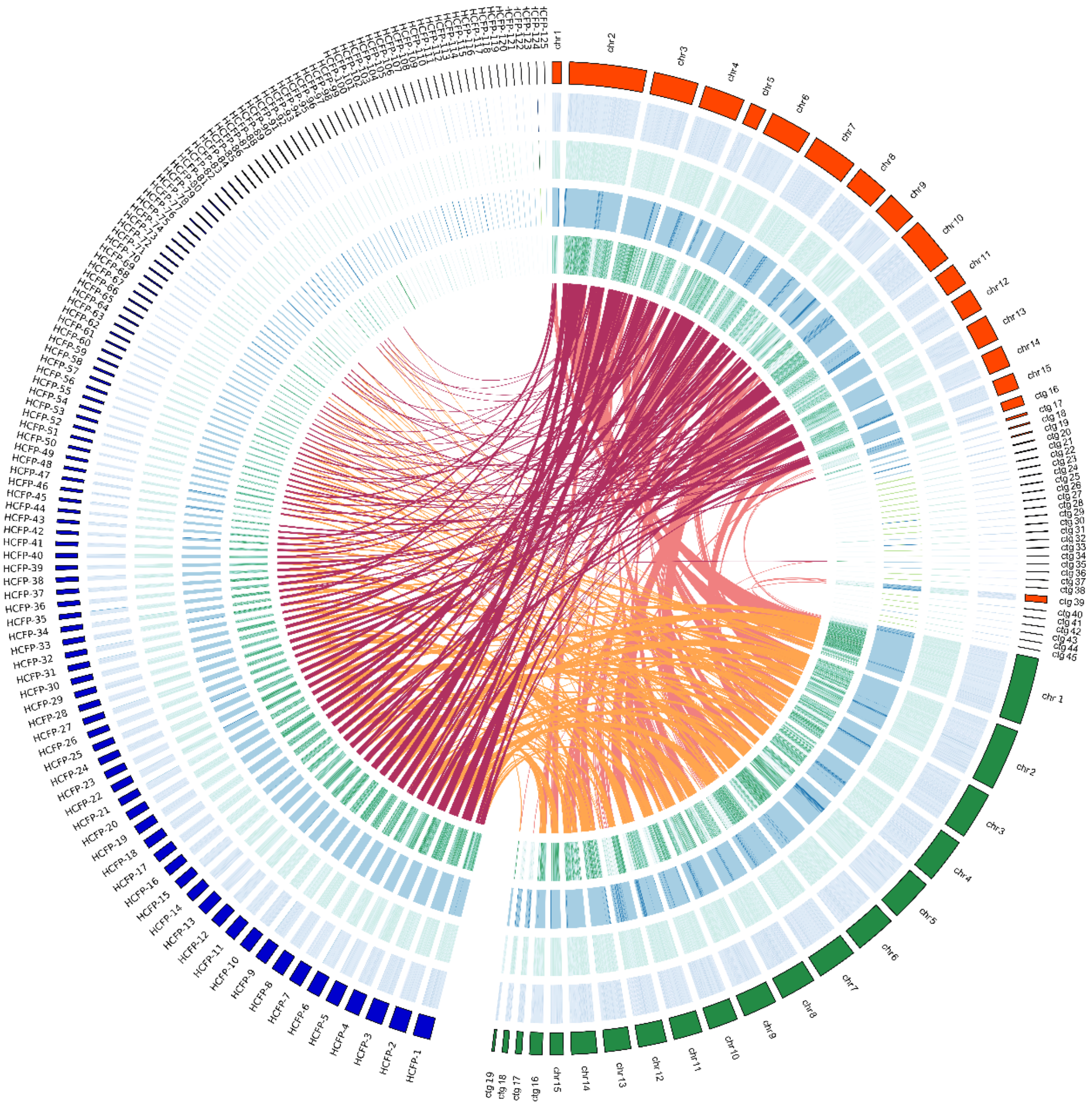

Figure S7. Comparative genome and genomic collinearity analysis among the genus *Hericium*.

From the outside to the inside are I. Chromosome and Contigs; II–IV. GC-density, GC-skew, AT-skew (window size 10 kb), V. Gene-density (window size 100 kb), VI. Whole-genome collinearity analysis based on protein-coding genes among the genus *Hericium*, the green color block refers to *H. rajendrae*, the red color block refers to *H. erinaceus*, and the blue color block refers to *H. coralloides*.

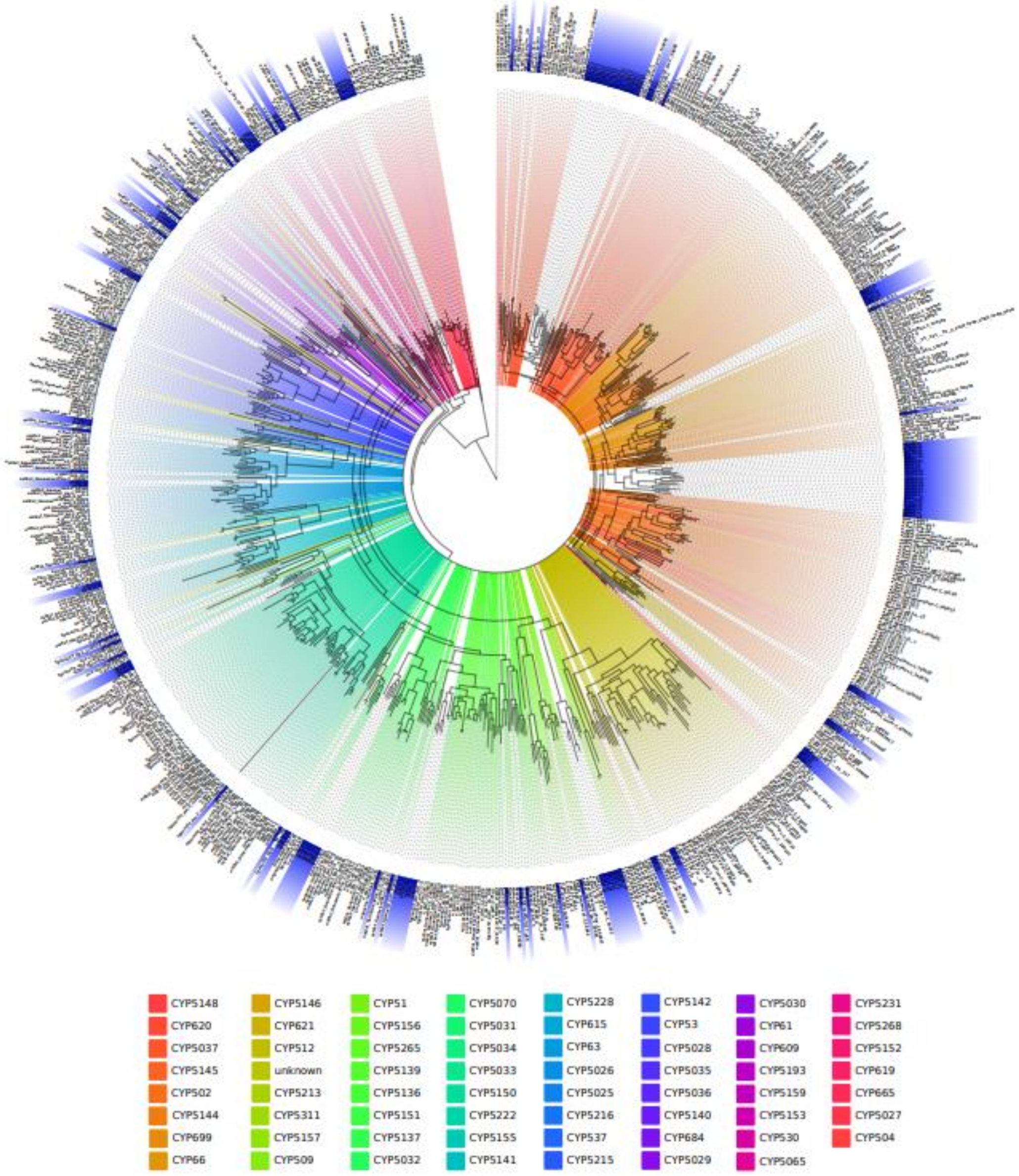

Figure S8. P450s Cluster analysis of the strain NPCB-A08 and other Basidiomycetes.

The sequences of the branch with color background and the corresponding sequence name without background are from Fungal Cytochrome P450 Database (http://passport.riceblast.snu.ac.kr/?t=P450). The background color of the branch corresponds to the category to which it belongs. The sequences with dark green background and corresponding branch without background are P450 sequences from the strain of NPCB-A08. Multiple sequence pairs are implemented with mafft V7 .505 (https://mafft.cbrc.jp/alignment/software/) with parameters --maxiterate 1000 --localpair. The evolutionary tree was constructed by IQtreeV2.2.3 with the parameters -m MFP -bb 1000 -alrt 1000 -abayes -nt AUTO[6].

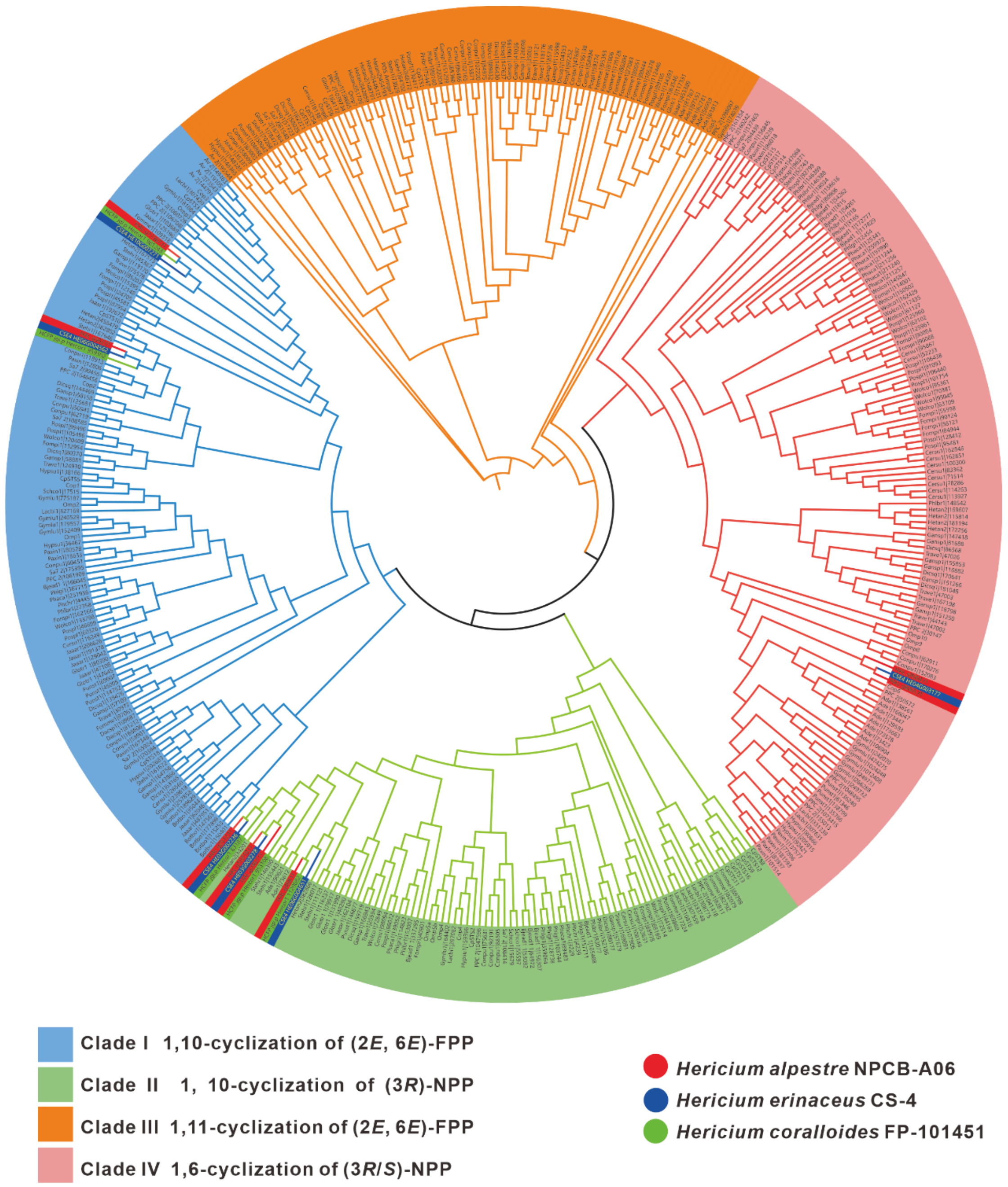

Figure S9. FPPs Cluster analysis of the strain NPCB-A08 and other Basidiomycetes.

The identified FPP sequences used for clustering were obtained from a reported literature[7]. Multiple sequence alignment and evolutionary tree construction were performed as described above.

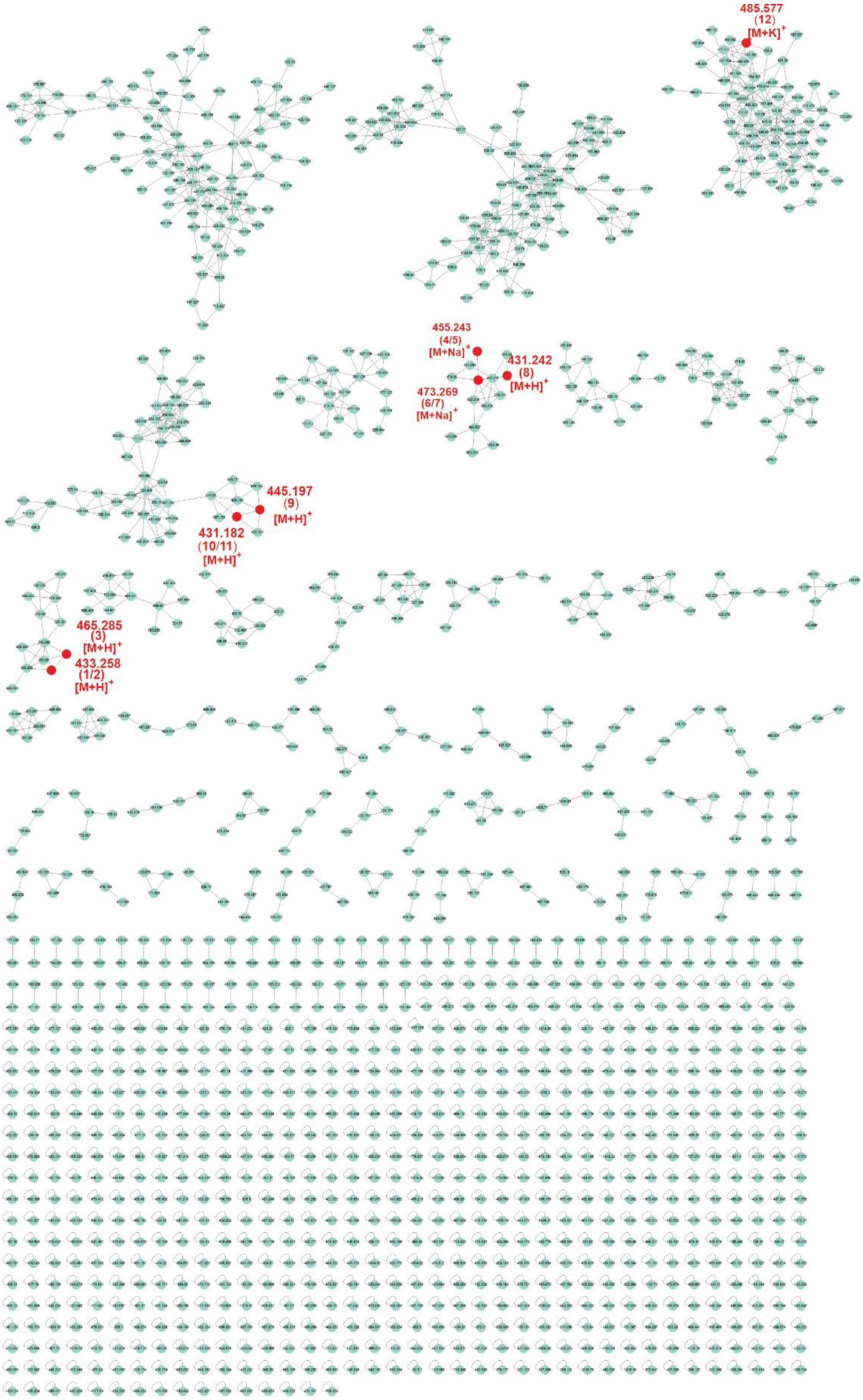

Figure S10. Molecular network analysis of metabolites from the mycelium and fruiting bodies of the strain NPCB-A08.

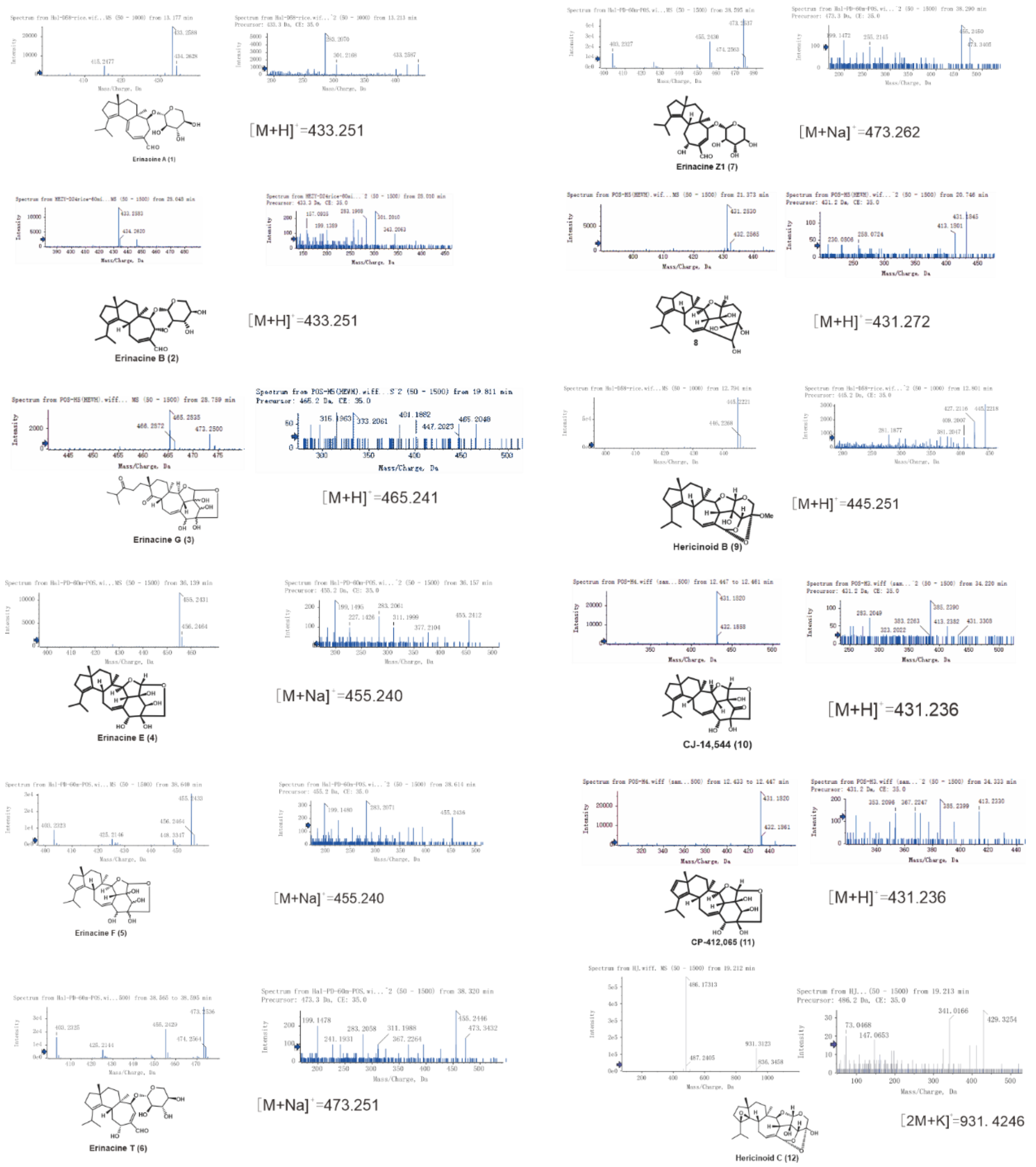

Figure S11. The LC-ESI-HRMS and LC-ESI-HRMS/MS spectrums of isolates from the strain NPCB A08.

Mass spectral data were acquired in positive ion mode. Figures A-L correspond one-to-one with compounds 1-12.

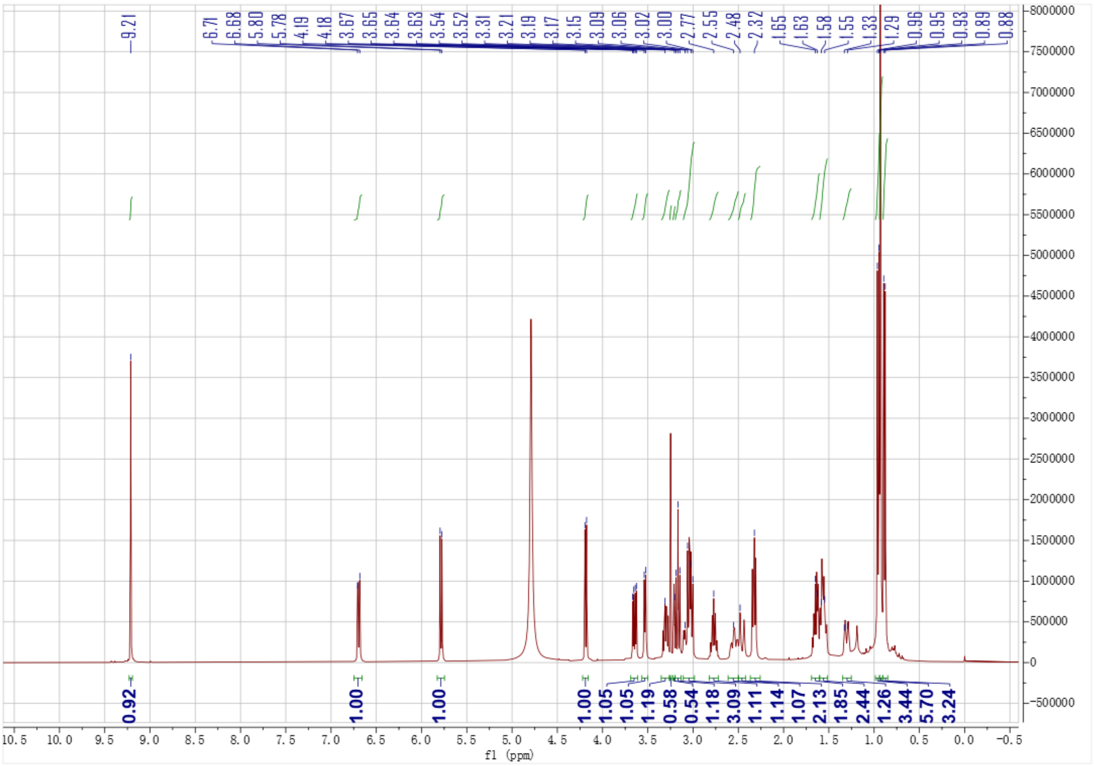
9.21
6.71
6.68
5.80
5.78
4.19
4.18
3.67
3.65
3.64
3.63
3.54
3.52
3.31
3.21
3.19
3.17
3.15
3.09
3.06
3.02
3.00
2.77
2.55
2.48
2.32
1.65
1.63
1.58
1.55
1.33
1.29
0.96
0.95
0.93
0.89
0.88
0.92
1.00
1.00
1.00
1.05
1.05
1.19
0.58
0.54
1.18
3.09
1.11
1.14
1.07
2.13
1.85
2.44
1.26
3.44
5.70
3.24
f1 (ppm)

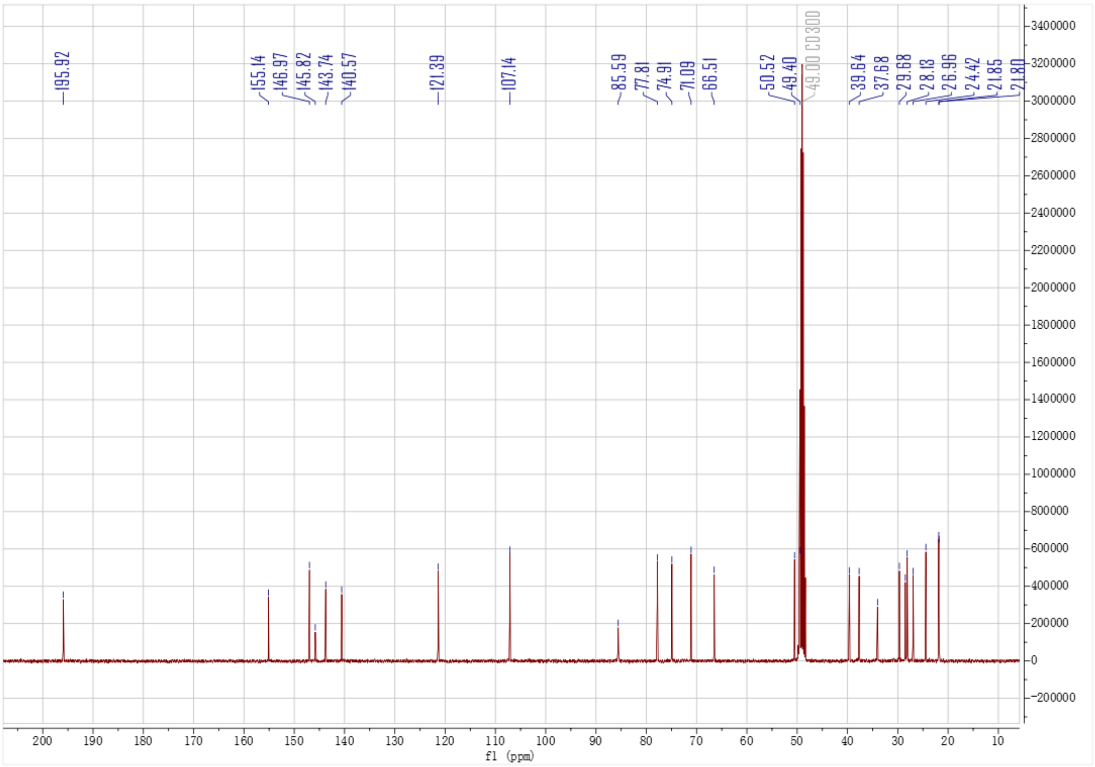
195.92
155.14
146.97
145.82
143.74
140.57
121.39
107.14
85.59
77.81
74.91
71.09
66.51
50.52
49.40
49.00 CD3OD
39.64
37.68
29.68
28.13
26.96
24.42
21.85
21.80
f1 (ppm)

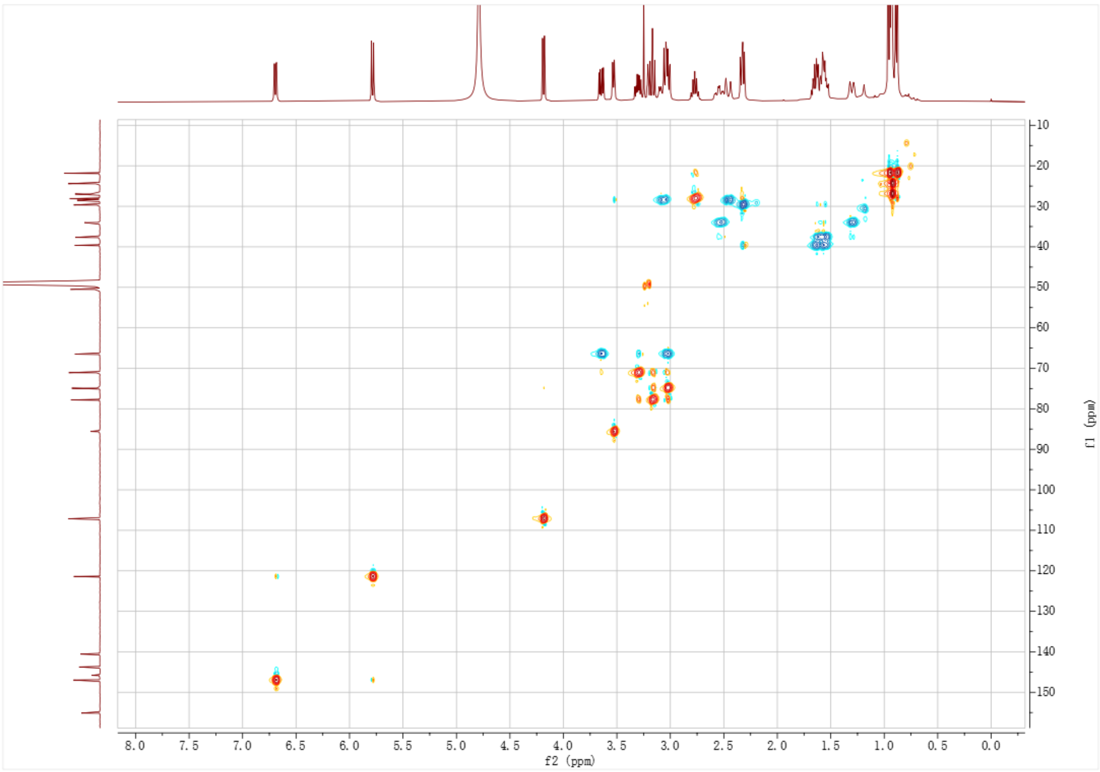
f2 (ppm)
f1 (ppm)

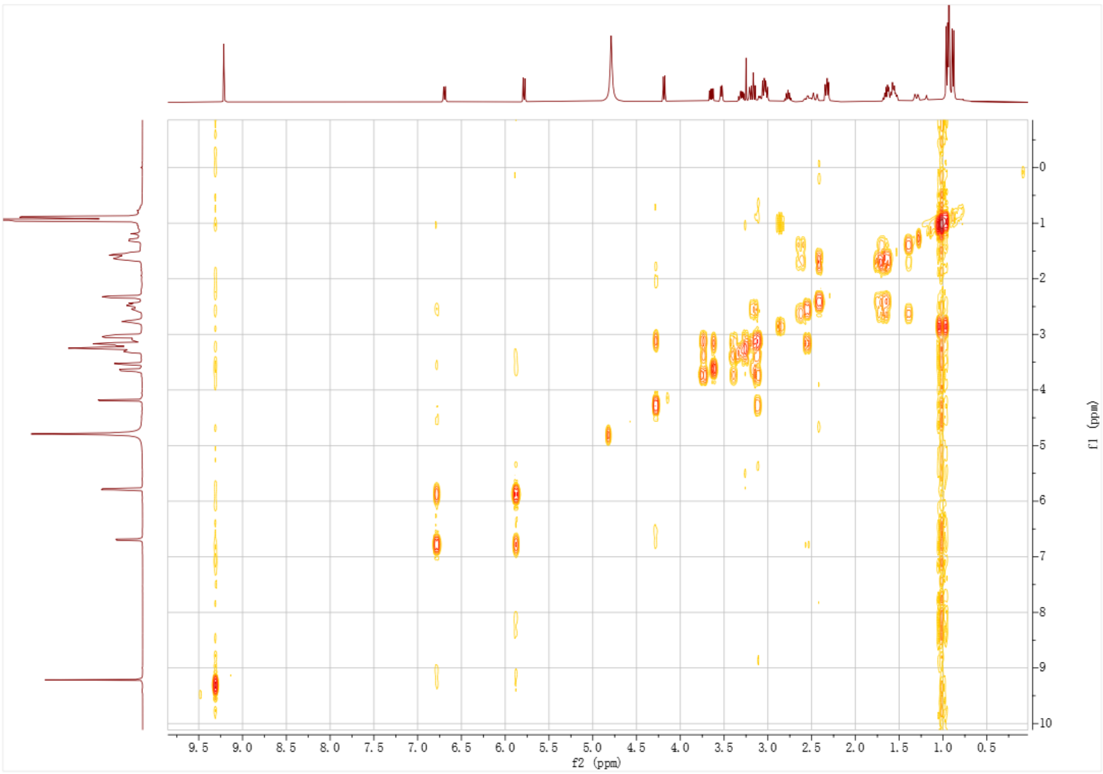
f2 (ppm)
f1 (ppm)

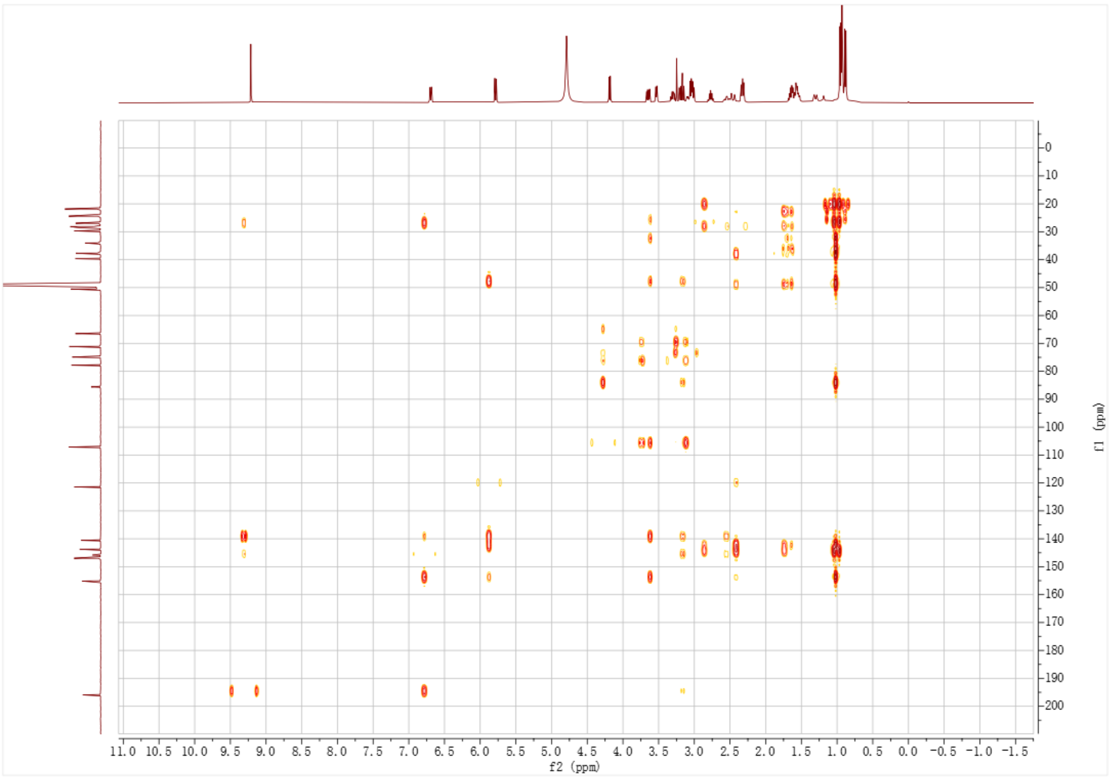

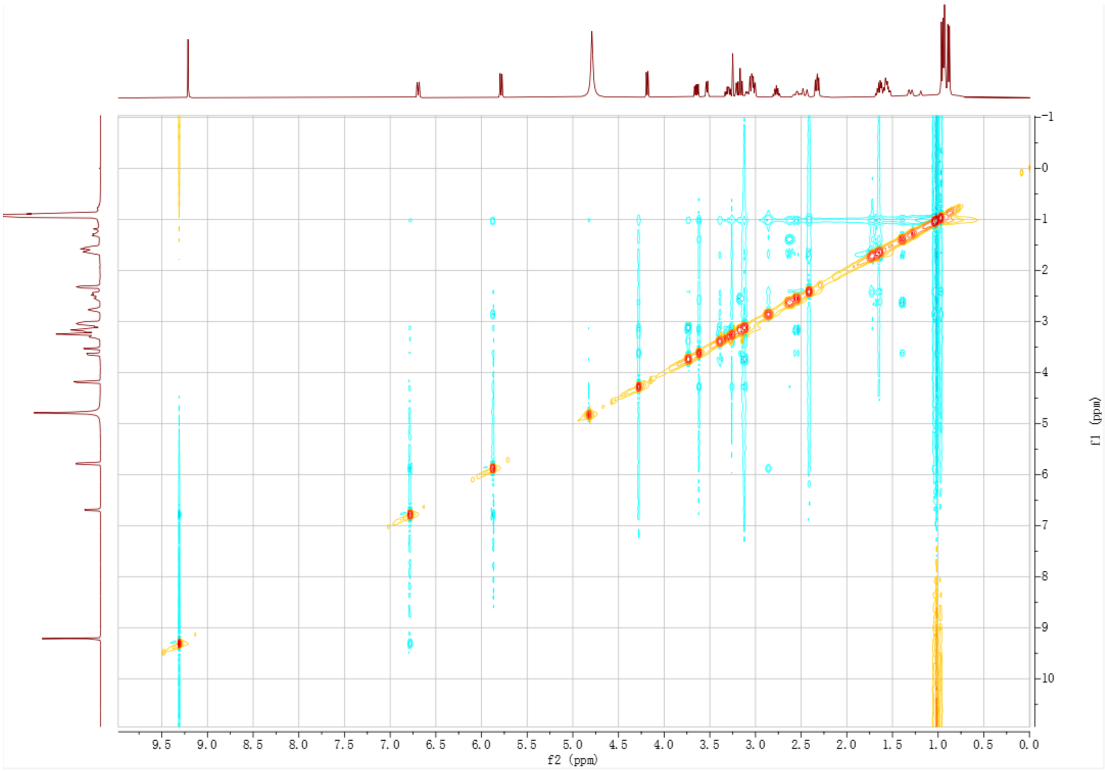

Figure S12. The NMR spectrums of compound 1 (MeOD) from the strain NPCB A08.

500 MHz for ^{1}H-NMR and 125 MHz for ^{13}C-NMR.

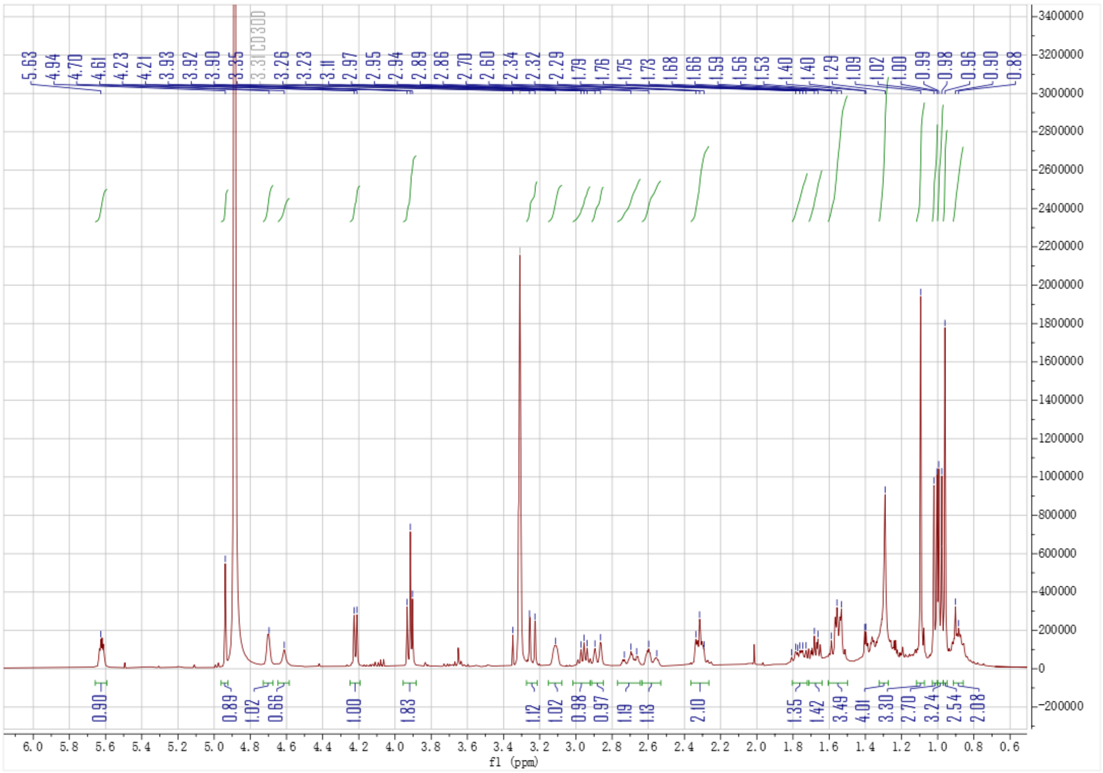

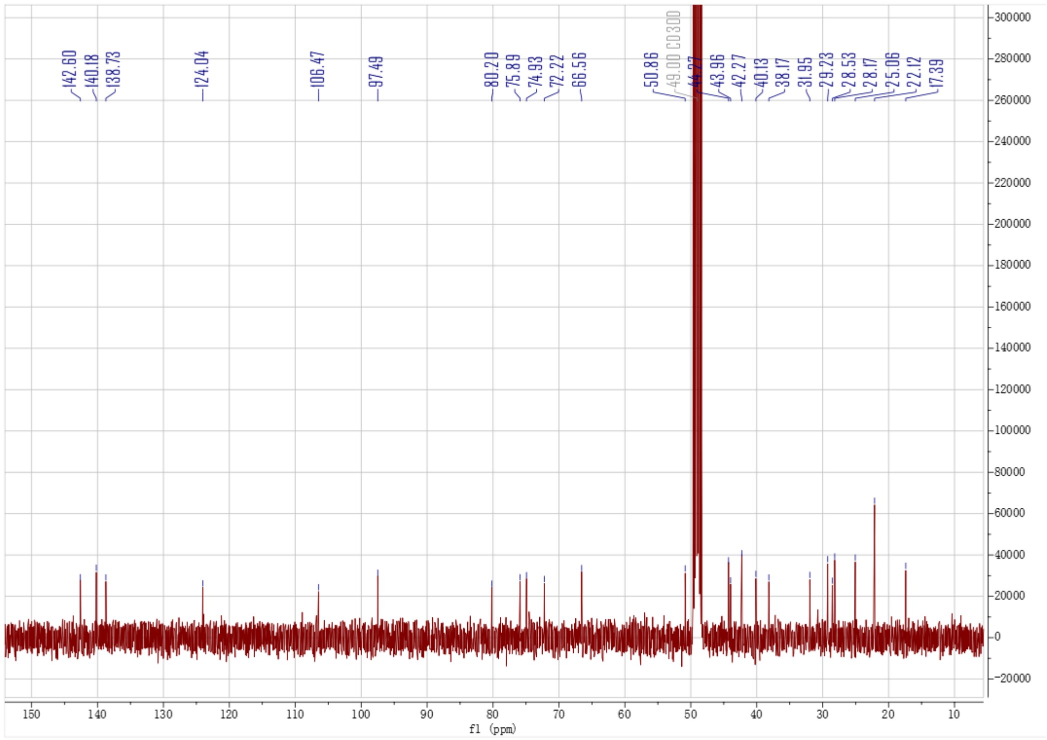

Figure S13. The NMR spectrums of compound 4 (MeOD) from the strain NPCB A08.

500 MHz for ^{1}H-NMR and 125 MHz for ^{13}C-NMR.

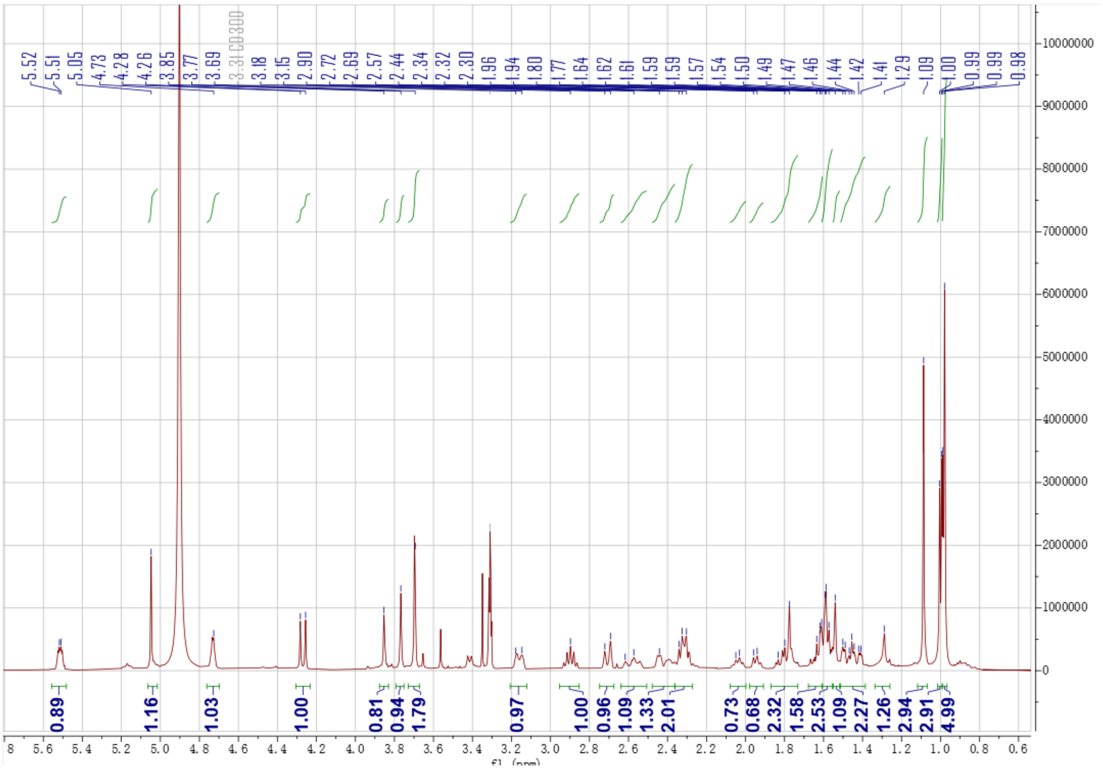

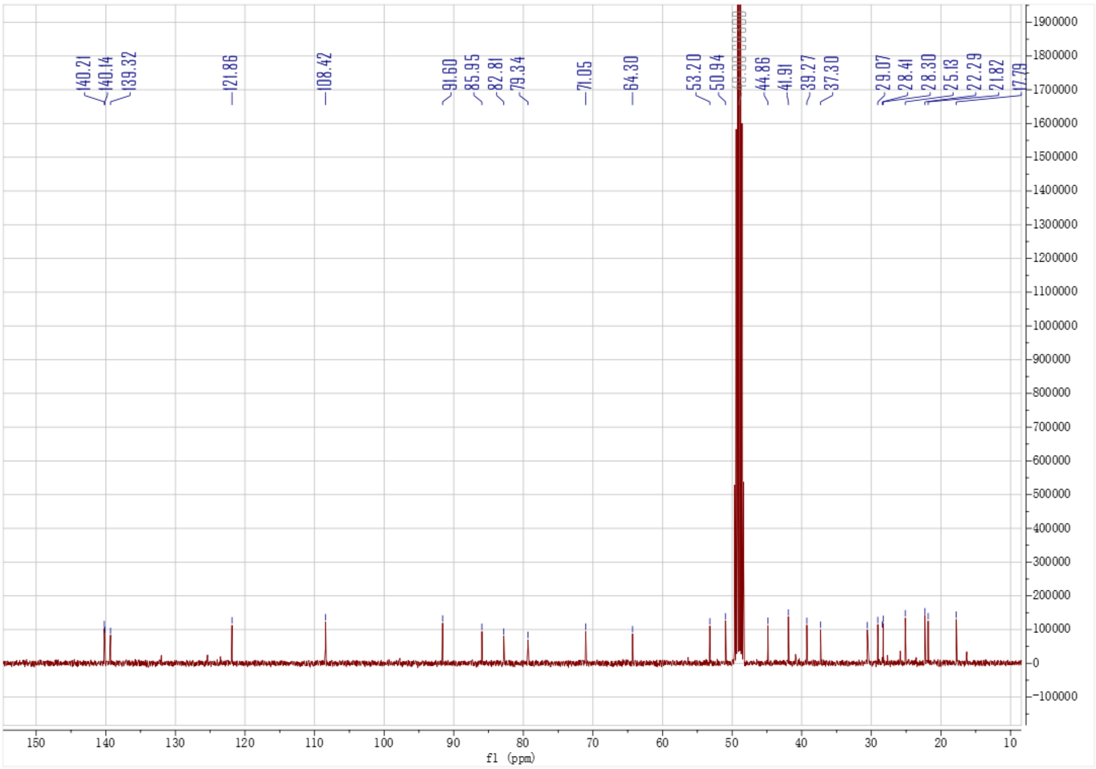

Figure S14. The NMR spectrums of compound 5 (MeOD) from the strain NPCB A08.

500 MHz for ^{1}H-NMR and 125 MHz for ^{13}C-NMR.

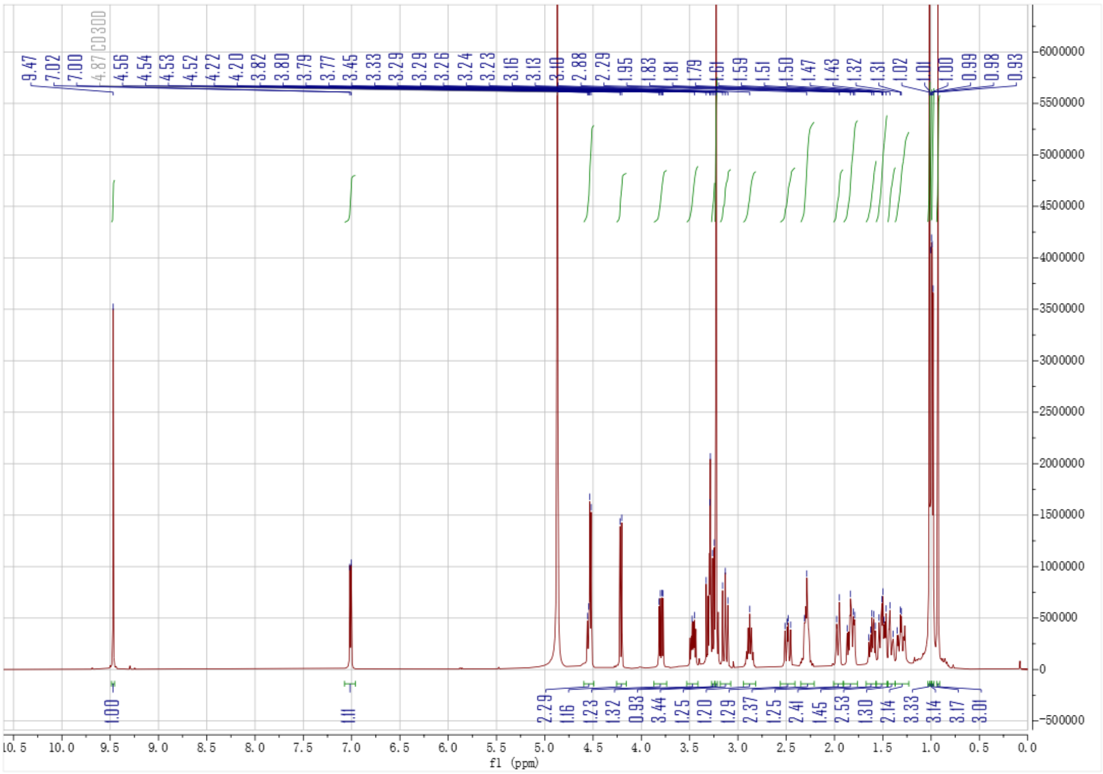

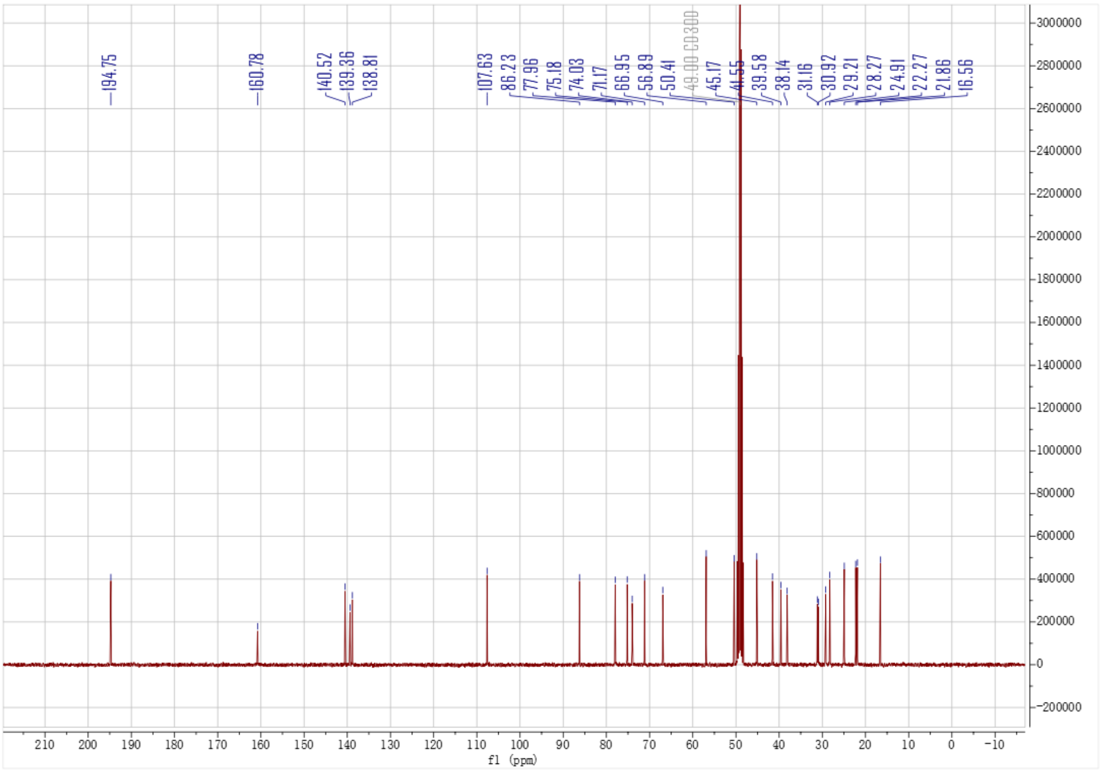

Figure S15. The NMR spectrums of compound 7 (MeOD) from the strain NPCB A08.

500 MHz for ^{1}H-NMR and 125 MHz for ^{13}C-NMR.

Reference

1. Kawagishi, H.; Shimada, A.; Shirai, R.; Okamoto, K.; Ojima, F.; Sakamoto, H.; Ishiguro, Y.; Furukawa, S. Erinacines A, B and C, strong stimulators of nerve growth factor (NGF)-synthesis, from the mycelia of Hericium erinaceum. *Tetrahedron Lett.* **1994**, *35*, 1569-1572.
2. Kawagishi, H.; Shimada, A.; Hosokawa, S.; Mori, H.; Sakamoto, H.; Ishiguro, Y.; Sakemi, S.; Bordner, J.; Kojima, N.; Furukawa, S. Erinacines E, F, and G, stimulators of nerve growth factor (NGF)-synthesis, from the mycelia of Hericium erinaceum. *Tetrahedron Lett.* **1996**, *37*, 7399-7402.
3. Wei, J.; Li, J.-y.; Feng, X.-l.; Zhang, Y.; Hu, X.; Hui, H.; Xue, X.; Qi, J. Unprecedented Neoverrucosane and Cyathane Diterpenoids with Anti-Neuroinflammatory Activity from Cultures of the Culinary-Medicinal Mushroom Hericium erinaceus. *Molecules* **2023**, *28*, 6380.
4. Chen, L.; Yao, J.-N.; Chen, H.-P.; Zhao, Z.-Z.; Li, Z.-H.; Feng, T.; Liu, J.-K. Hericinoids A−C, cyathane diterpenoids from culture of mushroom Hericium erinaceus. *Phytochem. Lett.* **2018**, *27*, 94-100.
5. Rupcic, Z.; Rascher, M.; Kanaki, S.; Köster, R.W.; Stadler, M.; Wittstein, K. Two New Cyathane Diterpenoids from Mycelial Cultures of the Medicinal Mushroom Hericium erinaceus and the Rare Species, Hericium flagellum. *Int. J. Mol. Sci.* **2018**, *19*, 740.
6. Minh, B.Q.; Schmidt, H.A.; Chernomor, O.; Schrempf, D.; Woodhams, M.D.; von Haeseler, A.; Lanfear, R. IQ-TREE 2: New Models and Efficient Methods for Phylogenetic Inference in the Genomic Era. *Molecular Biology and Evolution* **2020**, *37*, 1530-1534.
7. Wu, J.; Yang, X.; Duan, Y.; Wang, P.; Qi, J.; Gao, J.M.; Liu, C. Biosynthesis of Sesquiterpenes in Basidiomycetes: A Review. *Journal of Fungi* **2022**, *8*, 913.
